# Left Ventricular Non-compaction in a 40-Year-Old Male With Congenital Hydrocephalus

**DOI:** 10.7759/cureus.29546

**Published:** 2022-09-24

**Authors:** Charlotte Ritchie, Sara Albagoush

**Affiliations:** 1 Internal Medicine, Creighton University School of Medicine, Omaha, USA

**Keywords:** non ischemic cardiomyopathy, heart failure with reduced ejection fraction, congenital hydrocephalus, left ventricular non-compaction cardiomyopathy, left ventricular non compaction

## Abstract

Left ventricular non-compaction (LVNC) is a rare type of cardiomyopathy resulting from ineffective embryogenesis and is typically recognized shortly after birth. Here, we report a case of LVNC in a 40-year-old male with a history of congenital hydrocephalus who presented in overt heart failure. To date, there have been few reported cases of LVNC associated with hydrocephalus in the pediatric population and the pathophysiology is poorly understood. This nonclassical presentation of LVNC illustrates a rare cause of heart failure that may be related to hydrocephalus. Recognition of LVNC and further elucidating its association with hydrocephalus is crucial for identifying risk factors of LVNC and preventing its progression to heart failure.

## Introduction

Left ventricular eccentric hypertrophy is often characterized as a dilated cardiomyopathy, which is further classified into two groups: ischemic cardiomyopathy and non-ischemic cardiomyopathy. Ischemic cardiomyopathy is caused by myocardial infarction, leading to ischemia of the myocardial tissue and subsequent replacement of the myocytes by scar tissue. This scar tissue is unable to withstand the mechanical stress of the pumping heart and gradually stretches, leading to eccentric hypertrophy [1]. There are many causes of non-ischemic dilated cardiomyopathy including familial genetic mutations, autoimmune diseases, and various insults from pathogens and toxins. Left ventricular non-compaction (LVNC) is a rare type of cardiomyopathy that can also lead to dilation of the left ventricle. LVNC is also called hyper-trabeculation and results from a developmental error during embryogenesis. The consequence is a disruption in compaction during early development of the left ventricular myocardium and an increased number of endomyocardial trabeculations. Non-compaction is thought to result from a genetic mutation in 30-50% of cases and has been reported as part of certain congenital syndromes [2,3]. In the adult population, the prevalence has been reported to be 0.01-0.3% [4]. The phenotype ranges from a benign variant to severe cardiomyopathy with reduced ejection fraction, as in the patient case reported below.

## Case presentation

A 40-year-old male arrived at the emergency department after first presenting to an outside urgent care with complaints of heart palpitations and shortness of breath. He described two weeks of intermittent heart palpitations associated with epigastric pain radiating to his anterior chest as well as an unintentional weight gain of 10-15 pounds over the past month.

His past medical history was remarkable for asthma and idiopathic congenital hydrocephalus status post placement of a ventriculoperitoneal (VP) shunt 22 years prior. Family history was notable for hypertension and heart failure in both sides of his family. He denied a history of tobacco use and endorsed alcohol use of 2.5 alcohol units per week. 

**Figure 1:**
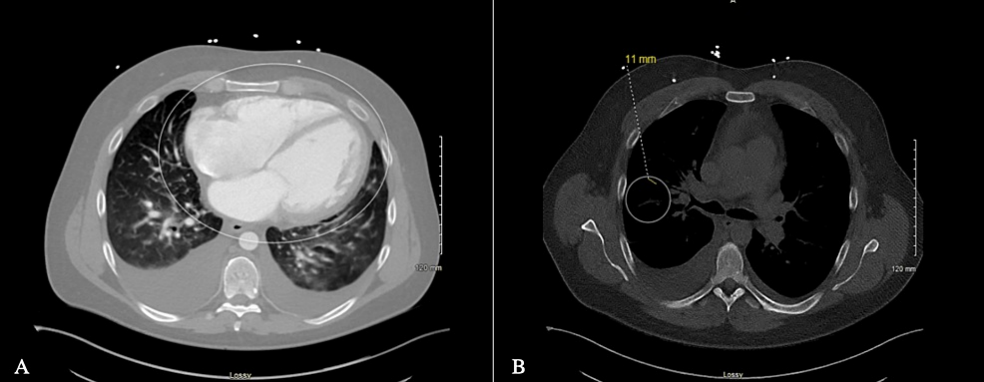
Computed tomography angiogram showing (A) moderate cardiomegaly, (B) 11 mm ground glass opacity in the upper lobe of the right lung

**Figure 2:**
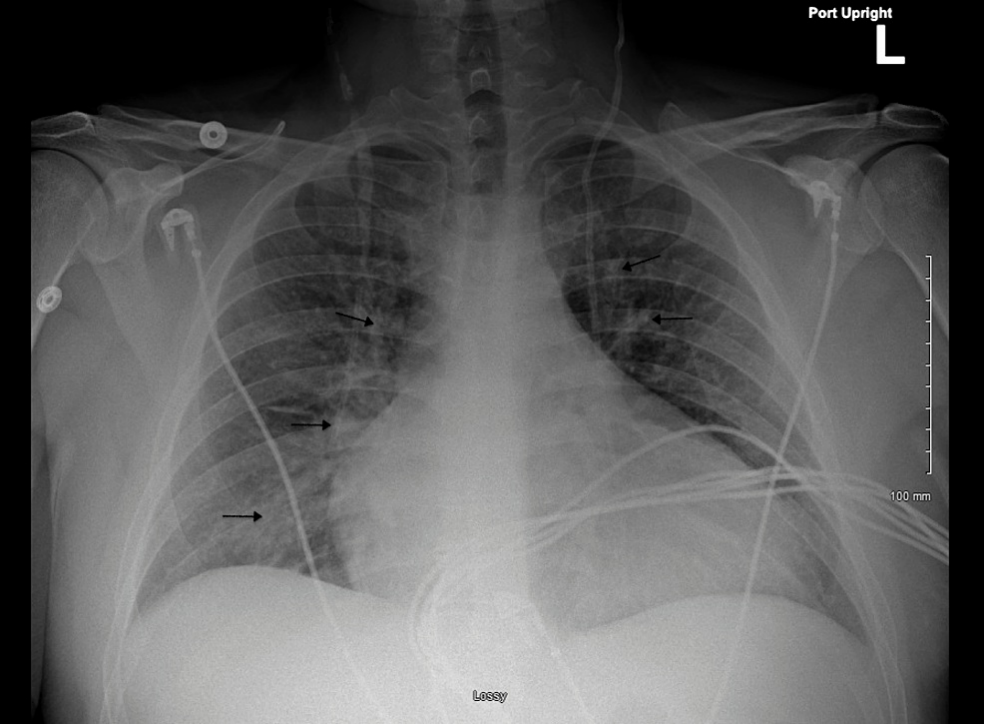
Chest x-ray showing interstitial pulmonary edema.

**Figure 3:**
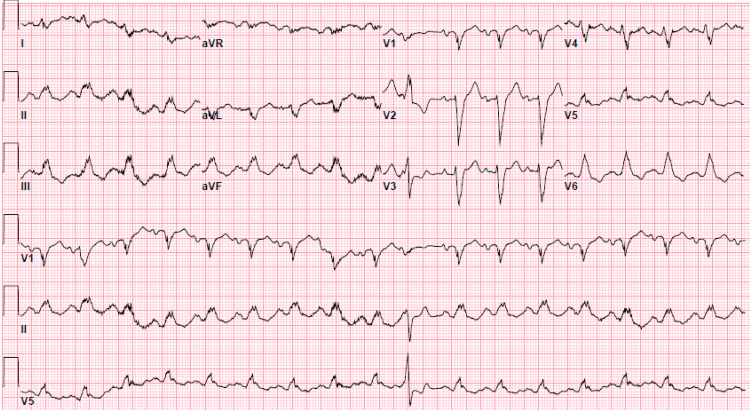
Electrocardiogram showing sinus tachycardia with premature ventricular contractions, right axis deviation, a left bundle branch block, and possible left atrial enlargement.

Upon arriving to the emergency department, the patient was afebrile and presented with a blood pressure of 175/107 mmHg and heart rate of 121 beats per minute. His labs were notable for an elevated troponin of 176.4 ng/mL and brain natriuretic peptide of 5759 pg/mL. Computed tomography angiogram of the abdomen and pelvis with and without contrast showed moderate cardiomegaly with evidence of right heart strain and a 11 mm ground glass opacity in the right upper lung lobe (Figure 1). A follow-up chest x-ray suggested interstitial pulmonary edema (Figure 2). Electrocardiogram was remarkable for left sinus tachycardia with frequent premature ventricular contractions, right axis deviation, a left bundle branch block, and possible left atrial enlargement (Figure 3). Physical examination was unremarkable. The patient was given furosemide, low-dose aspirin, heparin, and metoprolol and admitted to the hospital for further workup. 

Shortly after admission, an echocardiogram revealed an ejection fraction of 16% with severe left ventricular global hypokinesis and eccentric hypertrophy resulting in diastolic dysfunction and increased left atrial volume (>48 mL/m^2^). Right ventricular systolic pressure was elevated at 51.0 mmHg. Moderately reduced right ventricular systolic function and moderate mitral and tricuspid valve regurgitation were also reported.

**Figure 4:**
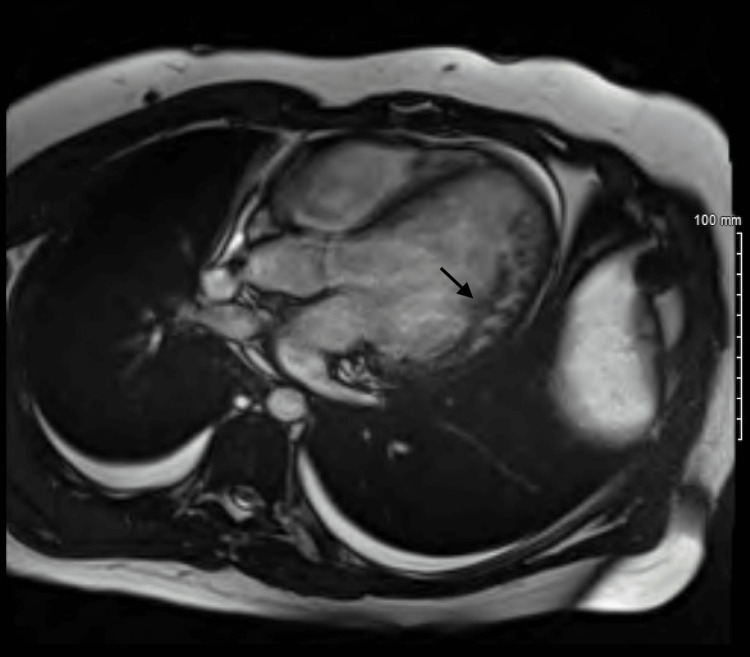
Cardiac magnetic resonance imaging (MRI) showing non-compaction of the left ventricle.

To further discern the cause of the patient's heart failure, left heart catheterization was subsequently done and revealed a left ventricular end-diastolic pressure of 35.0 mmHg but no evidence of discernible coronary artery disease. Cardiac magnetic resonance imaging (MRI) with late gadolinium enhancement was notable for prominent non-compaction of the myocardium at the anterolateral, lateral, and inferolateral left ventricle (Figure 4).

The hospital course was complicated by new onset atrial fibrillation. Although his CHA_2_DS_2_-VASc (congestive heart failure, hypertension, age ≥75 (doubled), diabetes, stroke (doubled), vascular disease, age 65 to 74, and sex category) score was low, he was started on anticoagulation with apixaban given that non-compaction is associated with stasis and blood clot formation. The patient was optimized by cardiology on guideline-directed medical therapy during hospitalization. He was discharged on metoprolol, spironolactone, empagliflozin, furosemide, apixaban, and sacubitril-valsartan. He was also provided with a LifeVest (a wearable defibrillator) upon discharge with instructions to follow up with his cardiologist weekly for medication titration and optimization.

## Discussion

This case describes a nonclassical presentation of overt systolic heart failure in a young male without any predisposing risk factors or clear etiology of his left ventricular eccentric hypertrophy. His workup was initially negative, and a clear cause of his dilated cardiomyopathy was unable to be discerned. The only apparent abnormality was not identified until cardiac MRI showed LVNC. Since the only irregularity identified during the patient’s hospitalization was LVNC, it is likely that this abnormality was the inciting cause of his heart failure. In 2014, a similar case was reported when a woman was admitted for congestive heart failure and found to have isolated ventricular non-compaction [4,5]. With improved diagnostic imaging techniques and wider access to their utilization, it is likely that the occurrence of these cases will increase.

This case is especially remarkable given that the patient was born with congenital hydrocephalus and subsequently treated with a VP shunt. A case of reversible LVNC has been reported during progressive hydrocephalus in pediatric populations [6]. The authors postulated that the catecholamine surge triggered by the stress of progressive hydrocephalus on the brain parenchyma resulted in LVNC, a pathophysiology similar to that of Takotsubo cardiomyopathy. However, it is unknown whether the cause of LVNC was related to the patient’s hydrocephalus given that his VP shunt was patent and placed years prior to his presentation. Nonetheless, there may be a more complex relationship not yet discerned between LVNC and hydrocephalus.

## Conclusions

Here, we illustrated a case of LVNC in an adult patient with no known congenital syndromic or genetic risk factors. Our case also identified that there may be a relationship between LVNC and hydrocephalus beyond the mechanisms previously proposed. However, with the underlying pathogenesis of LVNC being poorly understood, further research into possible etiologies and risk factors for LVNC development is vital to help elucidate the relationship between LVNC and hydrocephalus. Recognizing LVNC as a possible etiology for congestive heart failure is important as early detection of non-compaction is vital to preventing its inevitable progression to severe heart failure.
